# Surgical Risks Associated with Winter Sport Tourism

**DOI:** 10.1371/journal.pone.0124644

**Published:** 2015-05-13

**Authors:** Stéphane Sanchez, Cécile Payet, Jean-Christophe Lifante, Stéphanie Polazzi, François Chollet, Matthew J Carty, Antoine Duclos

**Affiliations:** 1 Hospices Civils de Lyon, Pôle Information Médicale Evaluation Recherche, Lyon, Rhône, France; 2 Université Claude Bernard Lyon 1, Faculté de médecine Lyon Est, Lyon, Rhône, France; 3 Hospices Civils de Lyon, Centre Hospitalier Lyon Sud-Service de Chirurgie Générale et Endocrinienne, Pierre Bénite, Rhône, France; 4 Université Claude Bernard Lyon 1, Faculté de médecine Lyon Sud, Lyon, Rhône, France; 5 Center for Surgery and Public Health, Brigham and Women's Hospital—Harvard Medical School, Boston, Massachusetts, United States of America; 6 Université Claude Bernard Lyon 1, EAM Santé-Individu-Société 4128, Lyon, Rhône, France; 7 Health Services and Performance Research Lab, Lyon, France; University of Florida College of Medicine, UNITED STATES

## Abstract

**Background:**

Mass tourism during winter in mountain areas may cause significant clustering of body injuries leading to increasing emergency admissions at hospital. We aimed at assessing if surgical safety and efficiency was maintained in this particular context.

**Methods:**

We selected all emergency admissions of open surgery performed in French hospitals between 2010 and 2012. After identifying mountain areas with increasing volume of surgical stays during winter, we considered seasonal variations in surgical outcomes using a difference-in-differences study design. We computed multilevel regressions to evaluate whether significant increase in emergency cases had an effect on surgical mortality, complications and length of stay. Clustering effect of patients within hospitals was integrated in analysis and surgical outcomes were adjusted for both patient and hospital characteristics.

**Results:**

A total of 381 hospitals had 559,052 inpatient stays related to emergency open surgery over 3 years. Compared to other geographical areas, a significant peak of activity was noted during winter in mountainous hospitals (Alps, Pyrenees, Vosges), ranging 6-77% volume increase. Peak was mainly explained by tourists’ influx (+124.5%, 4,351/3,496) and increased need for orthopaedic procedures (+36.8%, 4,731/12,873). After controlling for potential confounders, patients did not experience increased risk for postoperative death (ratio of OR 1.01, 95%CI 0.89-1.14, p = 0.891), thromboembolism (0.95, 0.77-1.17, p = 0.621) or sepsis (0.98, 0.85-1.12, p = 0.748). Length of stay was unaltered (1.00, 0.99-1.02, p = 0.716).

**Conclusion:**

Surgical outcomes are not compromised during winter in French mountain areas despite a substantial influx of major emergencies.

## Introduction

The French mountains are one of the top destinations for skiing and therefore generate an important tourist flow during the winter season [1]. Mass tourism in mountainous areas is associated with the accumulation of people experiencing trauma caused by winter sport activities [2,3,4,5]. Such clustering of injuries can lead to increased emergency admissions at hospitals due to influxes of patients requiring acute surgical—primarily orthopedic—care [6].

Traditionally, the advent of the winter sports season has been associated with increases in the volume of surgical procedures provided to both local and non-local populations. Injuries are typically witnessed most frequently in younger tourists who practice skiing or snowboarding with beginner or medium level experience at high speeds with new equipment and insufficient perception of the risk incurred [6,7,8,9]. Traumas tend to be more serious for non-local residents and usually affect the knees, shoulders and hands [10,11]. Severe head injuries can even cause life-threatening situations [12].

We assumed that an increased tourist influx coupled with high-risk injury cases [10] could saturate the emergency departments and disturb hospitals’ internal organization by exceeding bed capacity and available staff resources [13]. Previous studies suggest that hospital crowding can lead to higher risks of mortality [14,15,16], readmission [14,16], and adverse events [14,15,16] for patients with longer stays [14]. In this context, surgical teams’ ability to provide safe and efficient care can be impaired. Consequently, we investigated whether surgical outcomes were maintained during peak activity in French mountain hospitals. We used nationwide databases to determine the influence of tourist flow and winter sport practice on the volume of major emergency surgery and related mortality, complications and length of stay

## Materials and Methods

### Study design and population

A difference-in-differences study design was employed to evaluate whether emergency case volume increases associated with mass tourism in winter (December, January, February and March) had an effect on surgical outcomes [6,17,18]. This difference-in-differences analysis compared the change in outcome depending on seasons and areas and allowed us to account for seasonal and geographical unmeasured variations. First a control season (April, May, October and November) [6] without mass tourism in France was defined. Second, geographical areas with significant increasing volume of stays during winter were identified. Their volume of stays during winter was increased by at least 5% and greater than the 95% confidence interval compared to control season [19]. In this way, the difference-in-differences analysis allowed to evaluate whether observed changes in outcome between winter and control seasons were different between mountainous regions and other control areas.

All adults 18 years of age or older who underwent open surgery in France with at least one night spent at hospital were identified from January 2010 through December 2012. In order to preserve the homogeneity of the sample, ophthalmologic, dental and obstetric procedures were excluded as well as paediatric, ambulatory and palliative care, organ retrieval and those procedures with stays longer than 30 days. According to the above criteria, 6,327,762 hospital stays with surgical procedures performed in 1,186 hospitals were selected. We then identified a subgroup of emergency procedures reflecting all surgical specialties to enhance the generalizability of results across the spectrum of surgical care and hospitals. Finally, given our interest in monitoring surgical outcome variations over time and to minimize artifacts due to systematic coding errors, we further limited the study population to hospitals that provided continuous care over 3 years, where at least 50 emergency procedures were recorded per year and with a variation in the number of emergency procedures in less than 50% from one year to the other. After selecting care delivered during winter and control season, our final study population was composed of 559,052 inpatient stays in 381 facilities (S1 Fig).

### Data source and outcomes

The French nationwide hospitals database in acute care includes information about all inpatient stays that have occurred in every French public and private hospital with acceptable validity [20,21].Standard discharge abstracts for each of these hospitalizations contain compulsory information about the patient, primary and secondary diagnoses using the International Classification of Diseases, 10th revision (ICD-10 codes), as well as procedural codes associated with the care provided. In addition, the emergency status for each case at hospital admission is available.

The main outcome measures included in-hospital surgical mortality, complications and length of stay. In particular, two Patient Safety Indicators (PSI) were extracted from the database: the occurrence of postoperative pulmonary embolism or deep vein thrombosis (PSI 12) and the occurrence of postoperative sepsis (PSI 13) [22]. In order to adjust outcomes for case-mix variations using administrative data, our primary set of covariates included patient age, gender, patient’s residence code and 31 coexisting conditions extracted from the Elixhauser list of comorbidities [23]. The patient’s residence code permitted determination as to whether the patient was local or non-local relative to the treating institution. For every procedure, the anatomical site of the surgery was also considered and a more accurate description was provided for analyzing operations involving the musculoskeletal system. Additionally, to account for structural determinants, the facility type (public, private, or teaching hospital) was considered.

All legal conditions for epidemiological surveys were respected, and the French national commission governing the application of data privacy laws (the “Commission Nationale Informatique et Libertés”) issued approval for both projects. Since the study was strictly observational and used anonymous data, in accordance to the laws that regulate “non-interventional clinical research” in France, namely articles L.1121-1 and R.1121-2 of the Public Health Code (S1 Text), did not require the written informed consent from the participants or the authorization from any other ethics committee to conduct this survey.

### Statistical analysis

Difference-in-differences multilevel logistic regressions were used to model each surgical outcome (mortality and complications) taking into account the clustering effect of patients within hospitals [24]. An interaction term between season and area was included to consider the season effect. It was stated as a ratio of odds ratios (ROR) [19,25] for measuring the change in the odds ratios (OR) between mountainous areas and control areas. An ROR greater than 1.0 implied greater mortality and complications in a mountainous than in control areas during winter. Second, to model the length of stay, a difference-in-difference cox regression was used [26] [26]. Random effects (frailties) were included at the hospital level and were assumed to be gamma distributed [27] to take into account the clustering effect of patients within hospitals. In the same way, an interaction term between season and area was included and expressed as a ratio of hazard ratios (RHR). Furthermore, multilevel logistic and cox regressions were used to compare outcomes related to orthopedic procedures performed in mountainous areas during winter between tourists versus local residents. Data manipulation and analyses were performed using SAS software (version 9.2; SAS Institute Inc., Cary, NC).

### Ethics Statement

All legal conditions for epidemiological surveys were respected, and the French national commission governing the application of data privacy laws (the “Commission Nationale Informatique et Libertés”) issued approval for both projects. Since the study was strictly observational and used anonymous data, in accordance to the laws that regulate “non-interventional clinical research” in France, namely articles L.1121-1 and R.1121-2 of the Public Health Code, did not require the written informed consent from the participants or the authorization from any other ethics committee to conduct this survey.

## Results

### Population characteristics in the mountainous and control areas

**Fig 1:**
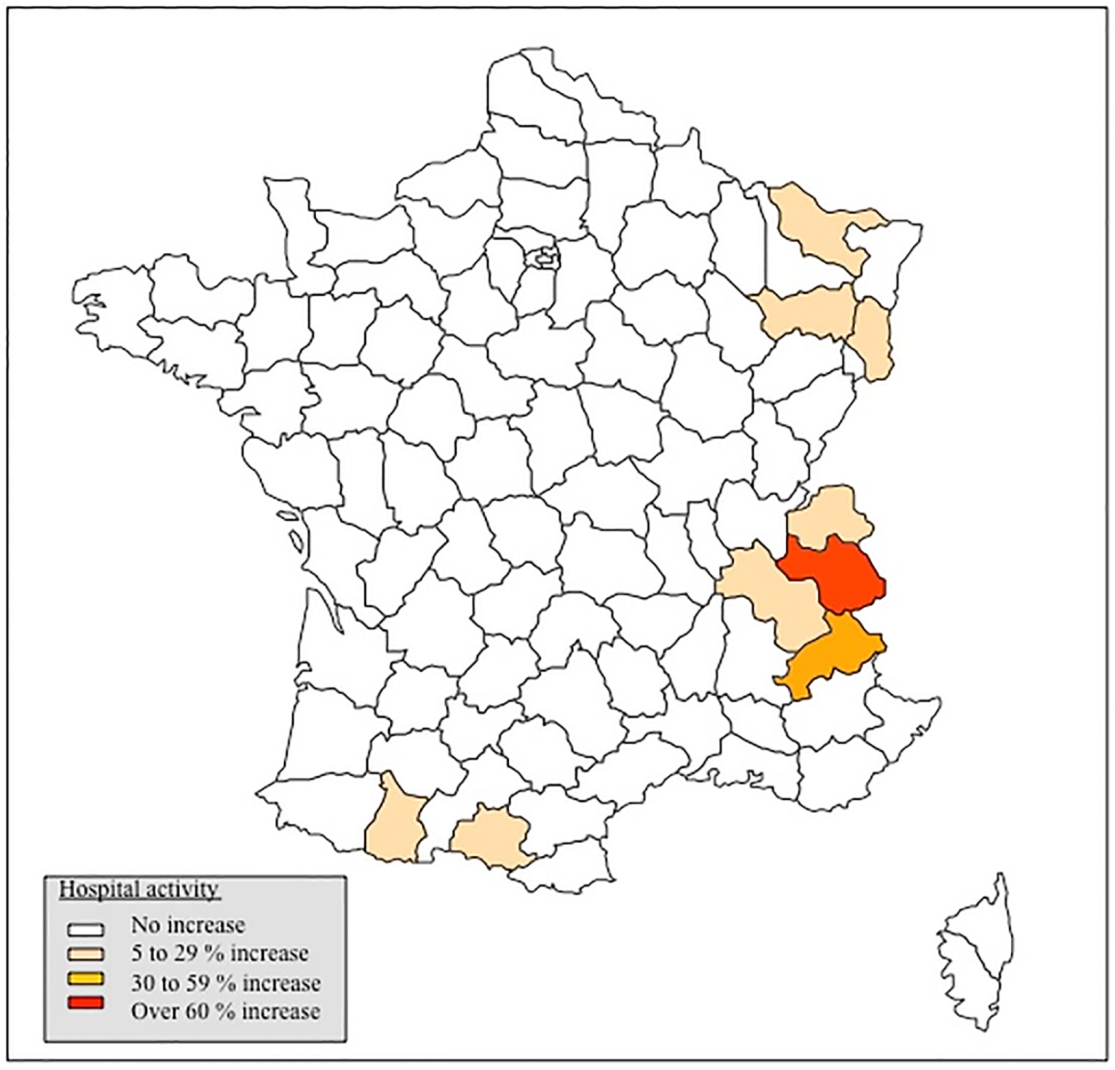
Variations in hospitals emergency admissions for open surgery during winter compared to control seasons. Variations in hospitals emergency admissions for open surgery during winter are depicted in a geographic representation. Three colours (light 5 to 29% increase; medium 30 to 59% increase; dark over 60% increase) identify geographical areas with significant increasing volume of stays during winter. Their volume of stays during winter was increased by at least 5% and greater than the 95% confidence interval compared to control season.

A total of 381 French hospitals had 559,052 inpatients stays related to open emergency surgery from 2010 to 2012 in winter and control season. Geographical areas having experienced a significant increase in the volume of emergency admission linked with open surgery procedures during winter are shown in Fig 1. These were mainly located in mountainous areas where tourists could practice winter sports. Activity increases were noted in 35 hospitals and ranged from 6% in Vosges to 15% in Pyrenees and as high as 77% in the Alps.

Hospitals were similar between mountainous and control areas (p = 0,796; Table 1). Admitted patients were more frequently tourists in mountainous (22.8%, 11,343/49,770) compared to control areas (17.1%, 87,296/509,282, p<0,001). Additionaly, surgical procedures involving the musculoskeletal system were more frequent in mountainous (61.2%, 30,477/49,770) compared to control areas (52.9%, 269,419/509,282, p<0,001), and especially related to lower limb and upper limb trauma.

**Table 1:** Characteristics of hospitals and population in the mountain and control areas.

Characteristics	Mountain areas	Control areas
Hospital	N = 35(%)	N = 346 (%)
Teaching	2 (5.7)	23 (6.6)
Public or Private non-for-profit	26 (74.3)	238 (68.8)
Private for profit	7 (20.0)	85 (24.6)
Inpatient stays	N = 49,770(%)	N = 509,282(%)
Women	24,903 (50.0)	253,639 (49.8)
Age (years), mean (SD)	58.4 (+/-22.6)	59.8 (+/-22.9)
No. comorbidities ^a^ mean (SD)	0.8 (+/-1.4)	0.9 (+/-1.3)
Patient’s origin		
Hospital area	38,427 (77.2)	421,986 (82.9)
French tourist	9,177 (18.4)	84,359 (16.6)
Non-french residents tourist	2,166 (4.4)	2,937 (0.6)
Surgical procedure codes		
Musculoskeletal system	30,477 (61.2)	269,419 (52.9)
Lower limb	18,796 (37.8)	160,088 (31.4)
Upper limb	8,940 (18.0)	74,665 (14.7)
Head, neck, trunk	2,503 (5.0)	30,608 (6.0)
Other	810 (1.6)	9,071 (1.8)
Digestive system	8,524 (17.1)	100,305 (19.7)
Operation on the Integumentary system	8,413 (16.9)	100,519 (19.7)
Cardiovascular system	2,351 (4.7)	35,079 (6.9)
Genital organs	1,178 (2.4)	15,204 (3.0)
Nervous system	870 (1.7)	13,009 (2.6)
Ear, Nose, Mouth and Pharynx	531 (1.1)	5,767 (1.1)
Respiratory system	174 (0.3)	2,659 (0.5)
Urinary system	135 (0.3)	2,064 (0.4)
Hematologic and Lymphatic system	116 (0.2)	1,673 (0.3)
Endocrine system	13 (0.0)	332 (0.1)
Surgical outcomes		
Inpatient mortality	1,305 (2.6)	15,956 (3.1)
Inpatient sepsis	961 (1.9)	11,219 (2.2)
Inpatient thromboembolism	393 (0.8)	3,368 (0.7)
Length of stay (days), mean (SD)	7.6 (+/-6.5)	8.0 (+/-6.8)

^a^ Elixhauser comorbidities include congestive heart failure, cardiac arrhythmias, valvular disease, pulmonary circulation disorders, peripheral vascular disorders, hypertension uncomplicated/complicated, paralysis, other neurological disorders, chronic pulmonary disease, diabetes uncomplicated/complicated, hypothyroidism, renal failure, liver disease, peptic ulcer disease excluding bleeding, AIDS/HIV, lymphoma, metastatic cancer, solid tumor without metastasis, rheumatoid arthritis/collagen vascular diseases, coagulopathy, obesity, weight loss, fluid and electrolyte disorders, blood loss anemia, deficiency anemia, alcohol abuse, drug abuse, psychoses, depression.

### Differences between winter and control seasons

Hospital emergency admissions increased during winter in mountainous areas, accounting for a total of 22.4% (5,012/22,379) additional stays for open surgery compared to control season (Table 2). This increase was observed in all facility types, as follow: +25.2% in public hospitals (4,053/16,062), +15.2% in teaching hospitals (681/4,469) and +15.0% in private hospitals (278/1,848). It was essentially driven by the influx of tourists (+124.5%, 4,351/3,496), both French (+101.8%, 3,095/3,041) and non-French resident (+276.0%, 1,256/455), while admission of patients living nearby the hospital remained stable (+3.5%, 661/18,883). Almost all the additional surgical procedures performed were related to the musculoskeletal system (+94.4%, 4,731/5,012). Corresponding activity increases during winter compared to control season was 36.8% (4,731/12,873), of which 40.1% (3,140/7,828) was attributable to lower limb procedures and 40.5% (1,506/3,717) to upper limb procedures. In particular, emergency admission increases for orthopaedic care were much higher among non-French resident people (+314.4%, 1,110/353) and French tourists (+154.5%, 2,788/1,804) than local residents (+7.8%, 833/10,679). Finally, admitted patients were younger with less comorbidities and crude surgical outcomes tended to improve during winter in mountainous area.

**Table 2:** Differences in characteristics of inpatient stays during winter and control seasons in the mountain and control areas.

	Mountain area & winter season	Mountain area & control season	Difference in mountain area	Control area & winter season	Control area & control season	Difference in control area
Hospital						
Teaching	5,150	4,469	681	67,053	67,120	-67
Public or Private non-for-profit	20,115	16,062	4,053	148,431	153,032	-4,601
Private for profit	2,126	1,848	278	36,304	37,342	-1,038
Inpatient stays	N = 27,391	N = 22,379	N = 5,012	N = 251,788	N = 257,494	N = -5,706
Women	13,668	11,235	2,433	126,513	127,126	-613
Men	13,723	11,144	2,579	125,275	130,368	-5,093
Comorbidities (average no.) ^a^	0,8	0,9	-0,1	0,9	0,9	0,0
Age (average years)	57,3	59,6	-2,3	60,3	59,4	0,9
Patient’s origin						
Hospital area	19,544	18,883	661	209,714	212,272	-2,558
French tourist	6,136	3,041	3,095	40,842	43,517	-2,675
Non-french residents tourist	1,711	455	1,256	1,232	1,705	-473
Surgical procedure codes						
Musculoskeletal system	17,604	12,873	4,731	134,832	134,587	245
Lower limb	10,968	7,828	3,140	81,165	78,923	2,242
Upper limb	5,223	3,717	1,506	36,734	37,931	-1,197
Head, neck, trunk	1,307	1,196	111	14,971	15,637	-666
Other	407	403	4	4,416	4,655	-239
Digestive system	4,280	4,244	36	48,936	51,369	-2,433
Integumentary system	4,194	4,219	-25	48,022	52,497	-4,475
Cardiovascular system	1,192	1,159	33	17,596	17,483	113
Genital organs	657	521	136	7,586	7,618	-32
Nervous system	463	407	56	6,506	6,503	3
Ear, Nose, Mouth and Pharynx	290	241	49	2,825	2,942	-117
Respiratory system	87	87	0	1,405	1,254	151
Urinary system	72	63	9	1,034	1,030	4
Hematologic and Lymphatic system	55	61	-6	859	814	45
Endocrine system	5	8	-3	175	157	18
Surgical outcomes						
Inpatient Mortality	691	614	77	8,327	7,629	698
Inpatient sepsis	466	495	-29	5,367	5,852	-485
Inpatient thromboembolism	198	195	3	1,695	1,673	22
Length of stay (average days)	7,3	7,8	-0,5	8,0	7,9	0,1

^a^ Elixhauser comorbidities include congestive heart failure, cardiac arrhythmias, valvular disease, pulmonary circulation disorders, peripheral vascular disorders, hypertension uncomplicated/complicated, paralysis, other neurological disorders, chronic pulmonary disease, diabetes uncomplicated/complicated, hypothyroidism, renal failure, liver disease, peptic ulcer disease excluding bleeding, AIDS/HIV, lymphoma, metastatic cancer, solid tumor without metastasis, rheumatoid arthritis/collagen vascular diseases, coagulopathy, obesity, weight loss, fluid and electrolyte disorders, blood loss anemia, deficiency anemia, alcohol abuse, drug abuse, psychoses, depression.

Regarding control area, there was a 2.2% (5,706/257,494) activity reduction during winter compared to control season. Relative seasonal differences in patients’ characteristics or surgical procedures were minimal and most outcomes remained stable.

### Difference in difference’s comparison of surgical outcomes

After adjusting for potential confounders, there was no difference between winter and control season in mountainous areas for all surgical outcomes (Table 3). Additionally, no differences were observed in comparison with control area. Patients did not experience higher risk for death (Ratio of Odds Ratio [ROR] 1.01, 95% confidence interval [95%CI] 0.89–1.14, p = 0.891), postoperative sepsis (ROR 0.98, 95%CI 0.85–1.12) or thromboembolism (ROR 0.95, 95%CI 0.77–1.17). Length of stay was unaltered (Ratio of Hazard Ratio 1.00, 95%CI 0.99–1.02).

**Table 3:** Comparison of risk-adjusted surgical outcomes during winter and control seasons between the mountain and control areas.

	Mountain area & winter season %	Mountain area & control season %	Odds Ratio^a^ ^c^ (IC 95%) in mountain area	Control area & winter season %	Control area & control season %	Odds Ratio^a^ ^c^ (IC 95%) in control area	Ratio of odds ratio^b^ ^c^ (IC 95%)
Mortality rate	2.5	2.7	1.10 (0.98 to 1.24)	3.3	3	1.09 (1.06 to 1.13)	1.01 (0.89 to 1.14)
Sepsis	1.7	2.2	0.89 (0.78 to 1.02)	2.1	2.3	0.91 (0.88 to 0.95)	0.98 (0.85 to 1.12)
Thromboembolism	0.7	0.9	0.95 (0.78 to 1.16)	0.7	0.7	1.00 (0.94 to 1.08)	0.95 (0.77 to 1.17)
Length of stay (days)^d^	7.3	7.8	1.00 (0.98 to 1.02)	8.0	7.9	1.00 (0.99 to 1.00)	1.00 (0.99 to 1.02)

^a^ “Control season” was the reference group in both groups.

^b^ The ratio of odds ratio compare the change in the complication and the mortality rates between two groups. A ratio of odds ratios greater than 1 suggests that the increase of complication and mortality from the “winter season” to control was greater in the “mountain area” group than in the “control area” group.

^c^ Adjusted for age, sex, comorbidities, patient’s residence, surgical procedures and type of facility.

^d^ Interpretation of hazard ratio and ratio of hazard ratio is the same than odds ratio and ratio of odds ratio.

Surgical outcomes were compared between tourists and local residents who underwent orthopaedic procedures during winter in mountain hospitals. Tourists had a lower mortality (OR 0.51, 95%CI 0.29–0.89), did not experience a higher risk for postoperative sepsis (OR, 0.51 95%CI 0.24–1.05), thromboembolism (OR 1.06, 95%CI 0.62–1.84) or extended length of stay (Hazard Ratio 1.01, 95%CI 0.97–1.06).

## Interpretation

### Main findings and comparison with other studies

In the context of winter tourism in French mountain areas, this evaluation provides evidence regarding the safety and efficiency of hospital surgical care. Every year, a substantial influx of patients needing major surgery is observed, primarily due to trauma linked with winter sport practice among tourists coming from other French areas or foreign countries. However, despite the threat of overcrowded healthcare facilities facing augmented volumes of major emergency cases, surgical outcomes remain unaltered. We observed no difference in mortality, postoperative complications and length of stay compared to control group related to other seasons and geographical areas.

The patterns of winter sport injuries have dramatically changed [28]. Since the 1970s, their incidence has dropped by half while upper extremity fractures and knees sprains became the most common injury types [29,30]. Our results are consistent with previous studies evidencing an increase in the volume of orthopaedic procedures performed during winter around ski resorts [5,6,8,9,10,11,31,32]. The observed peak in emergency admissions for open surgery was primarily associated with tourist populations. First, the more the tourist flow rises during winter, the more the volume of injuries needing surgical care tends to increase. Second, the probability of trauma occurrence is higher among tourists compared to local residents, as a potential consequence of the lack of experience and poor risk perception [6,7,8,9,10,11].

Faced with massive influxes of high risk cases admitted through emergency departments, mountain hospitals appeared to maintain surgical safety and efficient. Yet, previous works dealing with the issue of overcrowding emergencies have suggested its negative impact on healthcare quality and patients' related outcomes [14,15,16]. According to the “practices makes perfect” dogma, one reason might be that high volume surgical teams in mountainous areas are well trained for managing orthopaedic emergency cases and prepared for adapting to large seasonal variations in activity [33,34,35].

### Strengths and Limitations

Our study's strength relies on its utilization of an exhaustive nationwide sample of surgical care provided by all French facilities located in mountainous and other geographical areas. We opted for a difference-in-difference design allowing for control of potential confounders and secular trends using two control groups simultaneously. Over three successive years, seasonal variations in surgical outcomes between mountainous and other areas were compared, accounting for heterogeneity in patient case mix, clinical practices and data coding [36].

However, some methodological flaws relative to this large population-based study must be considered in interpreting findings. First, data were extracted from French administrative databases which have been initially implemented for establishing bills of inpatients stays. Accordingly, motivation of data coders is assumed to be influenced more by financial opportunism than epidemiological accuracy. Second, in order to maintain population representativeness, we assessed all types of surgical procedures based on a limited set of metrics. Thus, our macro-sample gathered a host of patients with strong heterogeneity in surgical risk and was not targeted for evaluating a few procedures based on specific complications and adjustment scheme [37,38].Third, regarding orthopaedic procedures, the granularity of available data was inadequate to know the potential reason for a ski injury or to control outcomes for every relevant covariates like care complexity or surgical staffing. Fourth, our statistics were focused on acute hospital care, not allowing monitoring of adverse events or patients recovery in the long-term.

### Conclusions and policy implications

This study suggests the need for implementing effective prevention strategy to reduce tourists' traumas during winter ski season. Seasonal increases in the number of bodily injuries occurring in mountainous areas among non-French residents and non-local residents is predictable every year. Avoiding winter sport related injuries through mass education programs might be of great importance to reduce its burden on healthcare facilities. Spreading recommendations on exposure to risk in mountains environment or adequate equipment to prevent severe injury may influence people behaviour towards wiser sports practices. Knowing how to manage trauma immediately before rescue services arrival may reduce their gravity and subsequently facilitate patient recovery. Towards this end, local authorities should target prevention campaign especially on tourists practicing winter sport activities [12,39,40]

Despite a massive increase in emergency case volumes, tourist influx during winter in mountainous areas does not appear to impact the French hospitals' outcomes regarding safety and efficiency of surgical care delivery. These findings may comfort French and European tourists who go skiing in the Alps and can benefit from a performing healthcare system. In order to preserve and keep improving the value of surgical processes, quality-improvement initiatives should be combined with optimal regulation of available resources over time depending on seasonal variations of the volume of procedures performed.

## Supporting Information

S1 Fig Flow diagram of hospitals and surgical cases retained in final dataset. This document contains our flowchart concern hospitals and surgical cases retained in final dataset.
(DOC)

S1 Text Legal laws. Laws that regulate “non-interventional clinical research” in France, namely articles L.1121-1 and R.1121-2 of the Public Health Code
(ZIP)
