# Supplementary material for: Surgical Risks Associated with Winter Sport Tourism
Source: PLoS One. 2015 May 13;10(5):e0124644. doi: 10.1371/journal.pone.0124644 (PMC4430272; doi:10.1371/journal.pone.0124644)
Supplement: S1 Fig — This document contains our flowchart concern hospitals and surgical cases retained in final dataset. (DOC) [file pone.0124644.s001.doc]

**Supporting information**

**S1 Fig.** Flow diagram of hospitals and surgical cases retained in final dataset

6,327,762 stays in 1,186 hospitals

962,748 stays in 654 hospitals

898,285 stays in 489 hospitals

886,926 stays in 405 hospitals

847,780 stays in 381 hospitals

Only emergency surgery

165 hospitals not providing continuously surgical care over 3 years

84 hospitals without at least 50 surgeries per year

24 hospitals with a variation in the number of emergency procedures in less than 50% from one year to the other

Selection of stays in winter season (November, December, January, February) and control season (September, October, March, April)

559,052 stays in 381 hospitals
