# Supplementary material for: Surgical Risks Associated with Winter Sport Tourism
Source: PLoS One. 2015 May 13;10(5):e0124644. doi: 10.1371/journal.pone.0124644 (PMC4430272; doi:10.1371/journal.pone.0124644)
Supplement: S1 Text — Laws that regulate “non-interventional clinical research” in France, namely articles L.1121-1 and R.1121-2 of the Public Health Code (ZIP) [file pone.0124644.s002.zip › Code de la santé publiqu...le L1121-1 _ Legifrance.pdf]

Liberté • Égalité • Fraternité
RÉPUBLIQUE FRANÇAISE
Legifrance.gouv.fr
LE SERVICE PUBLIC DE LA DIFFUSION DU DROIT

Chemin :

Code de la santé publique

- Partie législative
  - Première partie : Protection générale de la santé
    - Livre Ier : Protection des personnes en matière de santé
      - Titre II : Recherches biomédicales
        - Chapitre Ier : Principes généraux

Article L1121-1

Modifié par loi n°2006-450 du 18 avril 2006 - art. 31 JORF 19 avril 2006

Les recherches organisées et pratiquées sur l'être humain en vue du développement des connaissances biologiques ou médicales sont autorisées dans les conditions prévues au présent livre et sont désignées ci-après par les termes " recherche biomédicale ".

Les dispositions du présent titre ne s'appliquent pas :

1° Aux recherches dans lesquelles tous les actes sont pratiqués et les produits utilisés de manière habituelle, sans aucune procédure supplémentaire ou inhabituelle de diagnostic ou de surveillance ;

2° Aux recherches visant à évaluer les soins courants, autres que celles portant sur les médicaments, lorsque tous les actes sont pratiqués et les produits utilisés de manière habituelle mais que des modalités particulières de surveillance sont prévues par un protocole, obligatoirement soumis à l'avis du comité mentionné à l'article L. 1123-1. Ce protocole précise également les modalités d'information des personnes concernées. Les recherches ne peuvent être mises en oeuvre qu'après avis favorable, rendu dans un délai fixé par voie réglementaire, de l'un des comités de protection des personnes compétent pour le lieu où sont mises en oeuvre les recherches. La demande auprès du comité est faite par la personne physique ou morale qui prend l'initiative de ces recherches, en assure la gestion et vérifie que son financement est prévu. Lorsque les recherches portent sur des produits mentionnés à l'article L. 5311-1 à l'exception des médicaments, et figurant sur une liste fixée par arrêté du ministre chargé de la santé, pris sur proposition du directeur général de l'Agence française de sécurité sanitaire des produits de santé, le comité de protection des personnes s'assure auprès de l'Agence française de sécurité sanitaire des produits de santé que les conditions d'utilisation dans les recherches de ces produits sont conformes à leur destination et à leurs conditions d'utilisation courante.L'avis défavorable du comité mentionne, le cas échéant, que les recherches ne relèvent pas du présent 2°. Après le commencement des recherches, toute modification substantielle de celles-ci doit obtenir préalablement à leur mise en oeuvre un nouvel avis favorable du comité.

La personne physique ou la personne morale qui prend l'initiative d'une recherche biomédicale sur l'être humain, qui en assure la gestion et qui vérifie que son financement est prévu, est dénommée le promoteur. Celui-ci ou son représentant légal doit être établi dans la Communauté européenne. Lorsque plusieurs personnes prennent l'initiative d'une même recherche biomédicale, elles désignent une personne physique ou morale qui aura la qualité de promoteur et assumera les obligations correspondantes en application du présent livre.

La ou les personnes physiques qui dirigent et surveillent la réalisation de la recherche sur un lieu sont dénommées investigateurs.

Lorsque le promoteur d'une recherche biomédicale confie sa réalisation à plusieurs investigateurs, sur un même lieu ou sur plusieurs lieux en France, le promoteur désigne parmi les investigateurs un coordonnateur.

Liens relatifs à cet article

Cite:

Code de la santé publique - art. L1123-1 (V)
Code de la santé publique - art. L5311-1 (V)

Cité par:

Décret n°95-1172 du 6 novembre 1995 - art. 2 (Ab)
Arrêté du 16 août 2006 - art. 3 (V)
Décret n°2007-358 du 19 mars 2007 - art. 30 (V)
Arrêté du 9 mars 2007 - art. 2 (V)
Arrêté du 9 mars 2007 - art. 3 (V)
Arrêté du 5 mars 2008 - art. Annexe (Ab)

Arrêté du 5 mars 2008 - art., v. init.
Ordonnance n°2008-1305 du 11 décembre 2008 - art. 2, v. init.
Arrêté du 23 janvier 2009 - art. 2 (V)
Arrêté du 23 janvier 2009 - art. 2, v. init.
Arrêté du 23 janvier 2009 - art. 3 (V)
Arrêté du 23 janvier 2009 - art. 3, v. init.
Arrêté du 19 février 2009 (V)
Arrêté du 19 février 2009, v. init.
Arrêté du 19 février 2009 (V)
Arrêté du 19 février 2009, v. init.
Ordonnance n° 2010-49 du 13 janvier 2010 - art. 7 (V)
Arrêté du 13 janvier 2010 - art. Annexe au règlement intérieur (V)
Arrêté du 13 janvier 2010 - art., v. init.
Décision du 25 mai 2012, v. init.
LOI n°2013-442 du 30 mai 2013 - art. 8, v. init.
Décision du 12 février 2014 - art., v. init.
Code de la recherche - art. L223-1 (M)
Code de la recherche - art. L223-1 (M)
Code de la recherche - art. L223-1 (VD)
Code de la recherche - art. L223-1 (VT)
Code de la recherche - art. L344-3 (Ab)
Code de la recherche - art. L344-3 (VD)
Code de la santé publique - art. L1121-10 (VD)
Code de la santé publique - art. L1121-11 (VD)
Code de la santé publique - art. L1121-12 (VD)
Code de la santé publique - art. L1121-13 (VD)
Code de la santé publique - art. L1121-15 (V)
Code de la santé publique - art. L1121-15 (VD)
Code de la santé publique - art. L1121-15 (VT)
Code de la santé publique - art. L1121-16-1 (VD)
Code de la santé publique - art. L1121-16-3 (VD)
Code de la santé publique - art. L1121-3 (VD)
Code de la santé publique - art. L1121-4 (VD)
Code de la santé publique - art. L1121-5 (VD)
Code de la santé publique - art. L1121-6 (VD)
Code de la santé publique - art. L1121-7 (VD)
Code de la santé publique - art. L1121-8 (VD)
Code de la santé publique - art. L1121-8-1 (VD)
Code de la santé publique - art. L1121-9 (VD)
Code de la santé publique - art. L1122-1 (VD)
Code de la santé publique - art. L1122-1-1 (VD)
Code de la santé publique - art. L1122-1-3 (VD)
Code de la santé publique - art. L1123-10 (VD)
Code de la santé publique - art. L1123-6 (VT)
Code de la santé publique - art. L1123-7 (VD)
Code de la santé publique - art. L1123-8 (V)
Code de la santé publique - art. L1123-8 (V)
Code de la santé publique - art. L1123-8 (VD)
Code de la santé publique - art. L1123-9 (VD)
Code de la santé publique - art. L1125-1 (VD)
Code de la santé publique - art. L1125-3 (VD)
Code de la santé publique - art. L1126-10 (VD)
Code de la santé publique - art. L1126-5 (VD)
Code de la santé publique - art. L1221-8-1 (V)
Code de la santé publique - art. L1221-8-1 (VT)
Code de la santé publique - art. L1235-4 (VD)
Code de la santé publique - art. L1235-4 (VT)
Code de la santé publique - art. L1243-3 (M)
Code de la santé publique - art. L1243-3 (V)
Code de la santé publique - art. L1243-3 (V)
Code de la santé publique - art. L1243-3 (VT)
Code de la santé publique - art. L1245-4 (V)
Code de la santé publique - art. L1245-4 (VD)
Code de la santé publique - art. L1245-4 (VT)
Code de la santé publique - art. L1333-1 (VD)
Code de la santé publique - art. L1541-4 (V)
Code de la santé publique - art. L1541-4 (V)
Code de la santé publique - art. L1541-4 (VD)
Code de la santé publique - art. L1541-4 (VT)
Code de la santé publique - art. L5126-1 (M)
Code de la santé publique - art. R1121-1 (V)
Code de la santé publique - art. R1121-1 (V)
Code de la santé publique - art. R1121-2 (V)
Code de la santé publique - art. R1123-21 (M)
Code de la santé publique - art. R1123-21 (V)

Code de la santé publique - art. R1123-22 (V)
Code de la santé publique - art. R1123-22 (V)
Code de la santé publique - art. R1123-26 (V)
Code de la santé publique - art. R1211-12 (M)
Code de la santé publique - art. R1211-12 (V)
Code de la santé publique - art. R1233-1 (M)
Code de la santé publique - art. R1233-1 (V)
Code de la santé publique - art. R1235-3 (T)
Code de la santé publique - art. R1235-4 (T)
Code de la santé publique - art. R1235-4 (V)
Code de la santé publique - art. R1235-5 (V)
Code de la santé publique - art. R1242-1 (M)
Code de la santé publique - art. R1242-1 (M)
Code de la santé publique - art. R1242-1 (M)
Code de la santé publique - art. R1242-1 (V)
Code de la santé publique - art. R1243-1 (M)
Code de la santé publique - art. R1243-1 (V)
Code de la santé publique - art. R1243-57 (Ab)
Code de la santé publique - art. R1243-57 (V)
Code de la santé publique - art. R1243-63 (Ab)
Code de la santé publique - art. R1243-63 (V)
Code de la santé publique - art. R1243-70 (Ab)
Code de la santé publique - art. R1243-70 (V)
Code de la santé publique - art. R1245-15 (Ab)
Code de la santé publique - art. R1245-4 (V)
Code de la santé publique - art. R1245-8 (V)
Code de la santé publique - art. R4211-32 (V)
Code de la santé publique - art. R4321-66 (V)
Code de la santé publique - art. R5124-2 (M)
Code de la santé publique - art. R5124-2 (M)
Code de la santé publique - art. R5124-2 (V)
Code de la santé publique - art. R5124-2 (V)
Code de la santé publique - art. R5124-2 (V)
Code de la santé publique - art. R5124-2 (V)
Code de la santé publique - art. R5124-2 (V)
Code de la santé publique - art. R5124-3 (M)
Code de la santé publique - art. R5124-3-1 (V)
Code de la santé publique - art. R5211-37 (V)
Code de la santé publique - art. R5211-37 (V)
Code de la santé publique - art. R5211-37 (VD)
Code de la santé publique - art. R5313-6 (V)
Code de la santé publique - art. R5313-6 (V)
Code de la santé publique - art. R5313-6 (V)
Code de la santé publique - art. R6133-18 (V)
Code pénal - art. 223-8 (VD)
Code pénal - art. 723-1 (VD)

Codifié par:

Ordonnance 2000-548 2000-06-15
Loi 2002-303 2002-03-04 art. 92 JORF 5 mars 2002

Anciens textes:

Code de la santé publique - art. L209-1 (Ab)
Code de la santé publique - art. L209-1 (M)
