# Supplementary material for: Surgical Risks Associated with Winter Sport Tourism
Source: PLoS One. 2015 May 13;10(5):e0124644. doi: 10.1371/journal.pone.0124644 (PMC4430272; doi:10.1371/journal.pone.0124644)
Supplement: S1 Text — Laws that regulate “non-interventional clinical research” in France, namely articles L.1121-1 and R.1121-2 of the Public Health Code (ZIP) [file pone.0124644.s002.zip › Code de la santé publiqu...le L6113-7 _ Legifrance.pdf]

Chemin :

Code de la santé publique
- Partie législative
  - Sixième partie : Etablissements et services de santé
    - Livre Ier : Etablissements de santé
      - Titre Ier : Organisation des activités des établissements de santé
        - Chapitre III : Evaluation, accréditation et analyse de l'activité des établissements.

Article L6113-7

Modifié par Ordonnance n°2010-177 du 23 février 2010 - art. 4

Les établissements de santé, publics ou privés, procèdent à l'analyse de leur activité.

Dans le respect du secret médical et des droits des malades, ils mettent en oeuvre des systèmes d'information qui tiennent compte notamment des pathologies et des modes de prise en charge en vue d'améliorer la connaissance et l'évaluation de l'activité et des coûts et de favoriser l'optimisation de l'offre de soins.

Les praticiens exerçant dans les établissements de santé publics et privés transmettent les données médicales nominatives nécessaires à l'analyse de l'activité et à la facturation de celle-ci au médecin responsable de l'information médicale pour l'établissement dans des conditions déterminées par voie réglementaire après consultation du Conseil national de l'ordre des médecins.

Les praticiens transmettent les données mentionnées au troisième alinéa dans un délai compatible avec celui imposé à l'établissement.

Sous l'autorité des chefs de pôle, les praticiens sont tenus, dans le cadre de l'organisation de l'établissement, de transmettre toutes données concernant la disponibilité effective des capacités d'accueil et notamment des lits.A la demande du directeur, ce signalement peut se faire en temps réel.

Le praticien responsable de l'information médicale est un médecin désigné par le directeur d'un établissement public de santé ou l'organe délibérant d'un établissement de santé privé s'il existe, après avis de la commission médicale ou de la conférence médicale. Pour ce qui concerne les établissements publics de santé, les conditions de cette désignation et les modes d'organisation de la fonction d'information médicale sont fixés par décret.

Lorsque les praticiens appartenant au personnel des établissements publics de santé ne satisfont pas aux obligations qui leur incombent en vertu des troisième et quatrième alinéas, leur rémunération fait l'objet de la retenue prévue à l'article 4 de la loi de finances rectificative pour 1961 (n° 61-825 du 29 juillet 1961).

Liens relatifs à cet article

Cité par:

Loi n°78-17 du 6 janvier 1978 - art. 62 (V)
Loi n°78-17 du 6 janvier 1978 - art. 63 (V)
Loi n°99-1140 du 29 décembre 1999 - art. 33 (M)
Loi n°99-1140 du 29 décembre 1999 - art. 33 (V)
Décret n°2000-794 du 24 août 2000 - art. 6 (V)
Arrêté du 11 avril 2002 - art. 3 (Ab)
Arrêté du 11 avril 2002 - art. 3 (V)
Arrêté du 11 avril 2002 - art. 3 (V)
Arrêté du 11 avril 2002 - art. 3 (V)
Décret n°2002-960 du 4 juillet 2002 - art. 2 (V)
Décret n°2002-960 du 4 juillet 2002 - art. 6 (V)
Loi n°2003-1199 du 18 décembre 2003 - art. 34 (M)
Loi n°2003-1199 du 18 décembre 2003 - art. 34 (M)
Loi n°2003-1199 du 18 décembre 2003 - art. 34 (V)
Arrêté du 8 octobre 2004 - art. 3 (Ab)
Décret n°2004-1539 du 30 décembre 2004 - art. 6 (V)
Décret n°2004-1539 du 30 décembre 2004 - art. 6 (V)
Décret n°2004-1539 du 30 décembre 2004 - art. 7 (V)
Décret n°2004-1539 du 30 décembre 2004 - art. 7 (V)
Décret n°2004-1539 du 30 décembre 2004 - art. 7 (V)
Arrêté du 31 janvier 2005 - art. 1 (V)
Arrêté du 31 janvier 2005 - art. 2 (M)

Arrêté du 31 janvier 2005 - art. 2 (V)
Arrêté du 22 novembre 2006 - art. 8 (Ab)
Loi n°2006-1640 du 21 décembre 2006 - art. 77 (V)
Loi n°2006-1640 du 21 décembre 2006 - art. 77 (V)
Arrêté du 25 février 2008 - art. (V)
Arrêté du 25 février 2008 - art., v. init.
LOI n°2008-1330 du 17 décembre 2008 - art. 53, v. init.
LOI n°2008-1330 du 17 décembre 2008 - art. 59, v. init.
Arrêté du 30 novembre 2009 (V)
Arrêté du 30 novembre 2009, v. init.
Arrêté du 20 décembre 2010 (V)
Arrêté du 20 décembre 2010, v. init.
Arrêté du 14 décembre 2011 - art. 5 (V)
Arrêté du 14 décembre 2011 - art. 5, v. init.
Arrêté du 20 décembre 2011 (V)
Arrêté du 20 décembre 2011, v. init.
Arrêté du 20 décembre 2011 (V)
Arrêté du 20 décembre 2011, v. init.
Arrêté du 1er décembre 2011 - art. 3 (Ab)
Arrêté du 1er décembre 2011 - art. 3, v. init.
Arrêté du 11 juillet 2012 - art. 3 (Ab)
Arrêté du 11 juillet 2012 - art. 3, v. init.
Arrêté du 21 décembre 2012 (V)
Arrêté du 21 décembre 2012, v. init.
Décret n°2013-179 du 28 février 2013 - art. 1, v. init.
Arrêté du 4 mars 2013 (V)
Arrêté du 4 mars 2013, v. init.
Arrêté du 19 juillet 2013 - art. 3 (V)
Arrêté du 19 juillet 2013 - art. 3, v. init.
Arrêté du 19 décembre 2013 (V)
Arrêté du 19 décembre 2013, v. init.
Code de la santé publique - art. L6113-11 (V)
Code de la santé publique - art. L6122-19 (Ab)
Code de la santé publique - art. L6122-19 (M)
Code de la santé publique - art. L6145-1 (M)
Code de la santé publique - art. L6416-2 (V)
Code de la santé publique - art. R5126-53 (Ab)
Code de la santé publique - art. R6112-23 (V)
Code de la santé publique - art. R6112-23 (V)
Code de la santé publique - art. R6113-28 (V)
Code de la santé publique - art. R6113-28 (V)
Code de la santé publique - art. R6141-21 (Ab)
Code de la santé publique - art. R6141-21 (V)
Code de la santé publique - art. R6145-26 (V)
Code de la santé publique - art. R6145-26 (V)
Code de la santé publique - art. R6145-7 (V)
Code de la santé publique - art. R6145-7 (V)
Code de la santé publique - art. R6145-9 (T)
Code de la santé publique - art. R6146-21 (V)
Code de la santé publique - art. R6161-40 (V)
Code de la santé publique - art. R714-3-43 (Ab)
Code de la sécurité sociale. - art. L161-29 (V)
Code de la sécurité sociale. - art. L162-22-6 (V)
Code de la sécurité sociale. - art. L162-22-6 (V)
Code de la sécurité sociale. - art. L162-22-9 (M)
Code de la sécurité sociale. - art. L162-22-9 (MMN)
Code de la sécurité sociale. - art. L162-22-9 (V)
Code de la sécurité sociale. - art. L162-22-9 (V)
Code de la sécurité sociale. - art. L162-22-9 (V)
Code de la sécurité sociale. - art. L162-22-9 (V)
Code de la sécurité sociale. - art. R162-31-2 (V)
Code de la sécurité sociale. - art. R162-31-2 (V)
Code de la sécurité sociale. - art. R162-32 (M)
Code de la sécurité sociale. - art. R162-32-3 (M)
Code de la sécurité sociale. - art. R162-41 (M)
Code de la sécurité sociale. - art. R162-41 (M)
Code de la sécurité sociale. - art. R162-41-2 (V)
Code de la sécurité sociale. - art. R162-41-2 (V)
Code de la sécurité sociale. - art. R162-42-1 (V)
Code de la sécurité sociale. - art. R162-42-1 (V)
Code de la sécurité sociale. - art. R162-42-1-2 (V)
Code de la sécurité sociale. - art. R162-42-1-3 (V)

Anciens textes:

Code de la santé publique - art. L710-6 (Ab)
