# Supplementary material for: Surgical Risks Associated with Winter Sport Tourism
Source: PLoS One. 2015 May 13;10(5):e0124644. doi: 10.1371/journal.pone.0124644 (PMC4430272; doi:10.1371/journal.pone.0124644)
Supplement: S1 Text — Laws that regulate “non-interventional clinical research” in France, namely articles L.1121-1 and R.1121-2 of the Public Health Code (ZIP) [file pone.0124644.s002.zip › Code de la santé publiqu...le L6113-8 _ Legifrance.pdf]

Chemin :

Code de la santé publique

- Partie législative
  - Sixième partie : Etablissements et services de santé
    - Livre Ier : Etablissements de santé
      - Titre Ier : Organisation des activités des établissements de santé
        - Chapitre III : Evaluation, accréditation et analyse de l'activité des établissements.

Article L6113-8

Modifié par Ordonnance n°2010-177 du 23 février 2010 - art. 9 (V)

Les établissements de santé transmettent aux agences régionales de santé, à l'Etat ou à la personne publique qu'il désigne et aux organismes d'assurance maladie les informations relatives à leurs moyens de fonctionnement, à leur activité, à leurs données sanitaires, démographiques et sociales qui sont nécessaires à l'élaboration et à la révision du projet régional de santé, à la détermination de leurs ressources, à l'évaluation de la qualité des soins, à la veille et la vigilance sanitaires, ainsi qu'au contrôle de leur activité de soins et de leur facturation.

Les destinataires des informations mentionnées à l'alinéa précédent mettent en oeuvre, sous le contrôle de l'Etat au plan national et des agences au plan régional, un système commun d'informations respectant l'anonymat des patients, ou, à défaut, ne comportant ni leur nom, ni leur prénom, ni leur numéro d'inscription au Répertoire national d'identification des personnes physiques, et dont les conditions d'élaboration et d'accessibilité aux tiers, notamment aux établissements de santé publics et privés, sont définies par voie réglementaire.

Les établissements qui ne transmettent pas les informations mentionnées au premier alinéa dans les conditions et les délais fixés par voie réglementaire sont passibles d'une pénalité prononcée par le directeur général de l'agence régionale de santé , dans la limite de 5 % de leurs recettes annuelles d'assurance maladie.

Liens relatifs à cet article

Cite:

Code de la santé publique - art. L6115-2

Cité par:

Loi n°99-1140 du 29 décembre 1999 - art. 33 (V)
Décret n°2000-794 du 24 août 2000 - art. 6 (V)
Décret n°2002-960 du 4 juillet 2002 - art. 2 (V)
Décret n°2002-960 du 4 juillet 2002 - art. 6 (V)
Arrêté du 8 octobre 2004 - art. 3 (Ab)
Arrêté du 22 février 2008 (V)
Arrêté du 19 février 2009 - art. 1 (VD)
Arrêté du 10 février 2010, v. init.
Arrêté du 7 février 2011 (V)
Arrêté du 28 février 2011 - art. (V)
Arrêté du 20 décembre 2011, v. init.
Arrêté du 21 décembre 2012 (V)
Arrêté du 24 juillet 2013 (V)
Arrêté du 24 juillet 2013 - art. 1 (V)
Arrêté du 8 janvier 2014, v. init.
Arrêté du 14 février 2014 (V)
Code de la santé publique - art. Annexe 61-1 (Ab)
Code de la santé publique - art. L1431-2 (V)
Code de la santé publique - art. L6113-11 (V)
Code de la santé publique - art. L6122-19 (Ab)
Code de la santé publique - art. L6122-19 (M)
Code de la santé publique - art. R1414-7 (Ab)
Code de la santé publique - art. R6113-27 (V)
Code de la santé publique - art. R6113-28 (V)
Code de la santé publique - art. R6113-29 (V)
Code de la santé publique - art. R6113-33 (V)
Code de la santé publique - art. R6133-1 (M)
Code de la santé publique - art. R6133-1 (V)
Code de la santé publique - art. R6145-7 (V)

Code de la santé publique - art. R710-5-24 (Ab)
Code de la santé publique - art. R714-3-43 (Ab)
Code de la sécurité sociale. - art. L161-29 (V)
Code de la sécurité sociale. - art. L162-22-9 (V)
Code de la sécurité sociale. - art. R162-31-2 (V)
Code de la sécurité sociale. - art. R162-41-2 (V)

Anciens textes:
Code de la santé publique - art. L710-7 (Ab)
