# Supplementary material for: Surgical Risks Associated with Winter Sport Tourism
Source: PLoS One. 2015 May 13;10(5):e0124644. doi: 10.1371/journal.pone.0124644 (PMC4430272; doi:10.1371/journal.pone.0124644)
Supplement: S1 Text — Laws that regulate “non-interventional clinical research” in France, namely articles L.1121-1 and R.1121-2 of the Public Health Code (ZIP) [file pone.0124644.s002.zip › Code de la santé publiqu...le R1121-2 _ Legifrance.pdf]

Chemin :

Code de la santé publique
- Partie réglementaire
  - Première partie : Protection générale de la santé
    - Livre Ier : Protection des personnes en matière de santé
      - Titre II : Recherches biomédicales
        - Chapitre Ier : Principes généraux
          - Section 1 : Définitions.

Article R1121-2

Modifié par Décret n°2006-477 du 26 avril 2006 - art. 1 JORF 27 avril 2006

Les recherches définies au 1° de l'article L. 1121-1 appelées " recherches non interventionnelles " sont des recherches pour lesquelles la stratégie médicale destinée à une personne qui se prête à la recherche n'est pas fixée à l'avance par un protocole et relève de la pratique courante.

Les recherches non interventionnelles portant sur des produits mentionnés à l'article L. 5311-1 sont entendues comme toute recherche dans le cadre de laquelle le ou les produits sont prescrits ou utilisés de manière habituelle. Elles se conforment :

1° Pour les recherches portant sur les médicaments, à l'autorisation de mise sur le marché mentionnée à l'article L. 5121-8 ;

2° Pour les recherches portant sur les dispositifs médicaux, à la notice d'instruction, ou pour les recherches portant sur les dispositifs médicaux de diagnostic in vitro, à la notice d'utilisation ;

3° Pour les recherches portant sur les produits sanguins labiles, à la décision mentionnée au 1° de l'article L. 1221-8 ;

4° Pour les recherches portant sur les tissus issus du corps humain et sur les préparations de thérapie cellulaire, à l'autorisation mentionnée à l'article L. 1243-5 ;

5° Pour les recherches portant sur les produits cosmétiques ou les produits de tatouage, à toute étude menée chez des volontaires sains, à l'aide de méthodes d'investigations à risque négligeable, sur des produits dont la sécurité d'emploi est établie, lorsqu'ils sont appliqués dans des conditions normales d'emploi ou selon des méthodes reproduisant ces conditions.

La décision de prescription ou d'utilisation des produits mentionnés ci-dessus est indépendante de celle d'inclure dans le champ de la recherche la personne qui se prête à celle-ci.

Liens relatifs à cet article

Cite:
- Code de la santé publique - art. L1121-1 (VT)
- Code de la santé publique - art. L1221-8 (VT)
- Code de la santé publique - art. L1243-5 (MMN)
- Code de la santé publique - art. L5121-8 (V)
- Code de la santé publique - art. L5311-1 (VT)

Cité par:
- Code de la santé publique - art. R1121-4 (M)
- Code de la santé publique - art. R1121-7 (M)
- Code de la santé publique - art. R5121-178 (V)

Codifié par:
- Décret 2003-462 2003-05-21

Anciens textes:
- Code de la santé publique - art. R2047 (Ab)
